# First report of kyphoscoliosis in the narrow‐ridged finless porpoises (*Neophocaena asiaeorientalis*): Findings from congenital and degenerative cases comparison using post‐mortem computed tomography

**DOI:** 10.1002/vms3.1386

**Published:** 2024-03-08

**Authors:** Adams Hei Long Yuen, Sang Wha Kim, Kyunglee Lee, Young Min Lee, Sung Bin Lee, Min Ju Kim, Cherry Tsz Ching Poon, Won Joon Jung, Su Jin Jo, Mae Hyun Hwang, Jae Hong Park, Dasol Park, Sib Sankar Giri, Seung Hyeok Seok, Se Chang Park

**Affiliations:** ^1^ College of Veterinary Medicine and Research Institute for Veterinary Science Seoul National University Seoul Republic of Korea; ^2^ Radiotherapy and Oncology Centre Gleneagles Hospital Hong Kong Hong Kong SAR China; ^3^ Department of Microbiology and Immunology and Institute of Endemic Disease College of Medicine Seoul National University Seoul Republic of Korea; ^4^ College of Veterinary Medicine & Institute of Veterinary Science Kangwon National University Chuncheon Gangwon Republic of Korea; ^5^ Cetacean Research Institute National Institute of Fisheries Science Ulsan Republic of Korea; ^6^ Department of Surgery Queen Mary Hospital Pokfulam Hong Kong SAR China; ^7^ Department of Biomedical Sciences Seoul National University College of Medicine Seoul Republic of Korea; ^8^ Cancer Research Institute Seoul National University Seoul Republic of Korea

**Keywords:** anthropogenic activity, congenital kyphoscoliosis, degenerative kyphoscoliosis, narrow‐ridged finless porpoise, *Neophocaena asiaeorientalis*, post‐mortem computed tomography

## Abstract

**Introduction:**

Spinal deformities, including kyphoscoliosis, have been consistently documented in cetaceans. However, the majority of reported cases of kyphoscoliosis in cetaceans pertain to bottlenose dolphins, with limited information on its occurrence in narrow‐ridged finless porpoise (NFP) (*Neophocaena asiaeorientalis*).

**Materials and methods:**

In November 2021, two deceased NFPs were discovered stranded on the shores of the Republic of Korea. As part of the pioneer stranded cetacean imaging programme in the Republic of Korea, both carcasses underwent post‐mortem computed tomography (PMCT), revealing congenital and degenerative traumatic kyphoscoliosis, respectively.

**Results:**

Although kyphoscoliosis may not have directly caused the demise of these individuals, it is hypothesized that the reduced spinal range of motion and mobility associated with kyphoscoliosis may have contributed to their deaths.

**Conclusion:**

This case report presents the first documented cases of kyphoscoliosis in two NFPs stranded in Korean waters, utilizing PMCT as an efficient methodology for assessing skeletal abnormalities in cetaceans.

## Graphical abstract

This case report presents the first documented cases of kyphoscoliosis in two narrow‐ridged finless porpoises (*Neophocaena asiaeorientalis*) stranded in Korean waters. Both carcasses underwent post‐mortem computed tomography, revealing congenital kyphoscoliosis (CRI‐11873) and degenerative traumatic kyphoscoliosis (CRI‐11874).

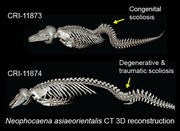

## 1 INTRODUCTION

Spinal deformities in mammals can be categorized as congenital, degenerative, traumatic or neuromuscular. Congenital kyphoscoliosis is a spinal deformity that originates during embryonic development (DeLynn et al., 2011). The aetiology of degenerative or traumatic scoliosis remains poorly understood, but it is generally believed to result from diseases (Kompanje, 1995) or physical trauma (Robinson, 2014). Although diverse causes of kyphoscoliosis in cetaceans have been reported, including congenital, degenerative and traumatic factors, these cases are predominantly associated with bottlenose dolphins (Berghan & Visser, 2000; Cobarrubia‐Russo et al., 2022; DeLynn et al., 2011; Morton, 1978; Robinson, 2014). Occasional reports have mentioned kyphoscoliosis or associated skeletal deformities in other *Delphinidae* species, such as killer whales (Berghan & Visser, 2000), Risso's dolphins (Nutman & Kirk, 1988), white‐beaked dolphins (Bertulli et al., 2015), long‐finned pilot whales (Sweeny et al., 2005), common dolphins and Hector's dolphins (Berghan & Visser, 2000). To date, there is a lack of published data on kyphoscoliosis in *Phocoenidae*, particularly in narrow‐ridged finless porpoise (NFP) (*Neophocaena asiaeorientalis*). NFPs are distributed across temperate and subtropical waters from the Taiwan Strait north into the Yellow Sea and into southern Japan (Jefferson & Wang, 2011). This species has a recorded maximum lifespan of 23 years (Kasuya, 1999). Although dietary variation was detected in different colonies of NFPs, crustaceans, fish and cephalopods were identified as their common prey organisms (Lu et al., 2016; Shirakihara et al., 2008).

Kyphoscoliosis in cetaceans is typically reported through in situ visual assessments during photo‐identification field studies (Bertulli et al., 2015), and more subtle abnormalities are discovered during necroscopic examinations (DeLynn et al., 2011). As part of the pioneering stranded cetacean imaging programme in the Republic of Korea, NFPs stranded in Korean waters have been routinely assessed using post‐mortem computed tomography (PMCT) to provide initial screening and supplementary evidence for conventional necropsy (Yuen et al., 2022, 2023). This case report presents the first documented cases of congenital, degenerative and traumatic kyphoscoliosis in two NFPs using PMCT.

## 2 CASE REPORT

In November 2021, one calf and one adult NFPs, hereinafter referred to as CRI‐11873 and CRI‐11874, respectively, were discovered stranded on the coasts of Busan (Nam‐gu, Busan) and Pohang (Nam‐gu, Pohang, Gyeongsangbuk‐do), Republic of Korea, respectively. Both NFP carcasses were in good condition [code 2 per Smithsonian Institution criteria (Geraci & Lounsbury, 2005)]. The age of the carcasses was estimated based on their total body length with reference to Lee et al. (2013). To minimize autolysis, the carcasses were promptly transported to the National Cetacean Research Institute (CRI), Republic of Korea, and stored at −20°C upon their initial report. Ethical approval was waived for this case report as it did not involve living or foetal subjects.

PMCT scans were conducted at the College of Veterinary Medicine, Kyungpook National University, using a Toshiba 16‐row multislice spiral CT scanner Alexion (Canon Medical Systems). The PMCT examinations were performed at 120 kVp, 150 mAs, with a section thickness of 0.8 mm. To encompass the entire body girth within the field of view (FOV), the FOV was set at 350 and 430 mm for CRI‐11873 and CRI‐11874, respectively. PMCT data set was reconstructed and assessed using Digital Imaging and Communications in Medicine (DICOM) viewer, Horos version 3.3.6. PMCT images were then reconstructed for three dimensional (3D) volume‐rendered images by accumulating the sequential transaxial data using a high spatial resolution algorithm inbuilt with Horos DICOM viewer. Bone density of the affected vertebrae was measured on PMCT multiplanar reconstructed sagittal images using Hounsfield unit (HU) quantification. All PMCT procedures were executed under the guidance of a registered radiographer (AHLY). Subsequent necroscopic examinations were carried out at a research centre in the Republic of Korea by board‐certified veterinarians (SWK and KL). All the specimens from the necropsy, including vertebral column and parasite specimens, were lodged at the research institute.

## 3 CRI‐11873 (FEMALE; 104.5 CM; 17.4 KG) (FIGURE 1a)

**FIGURE 1:**
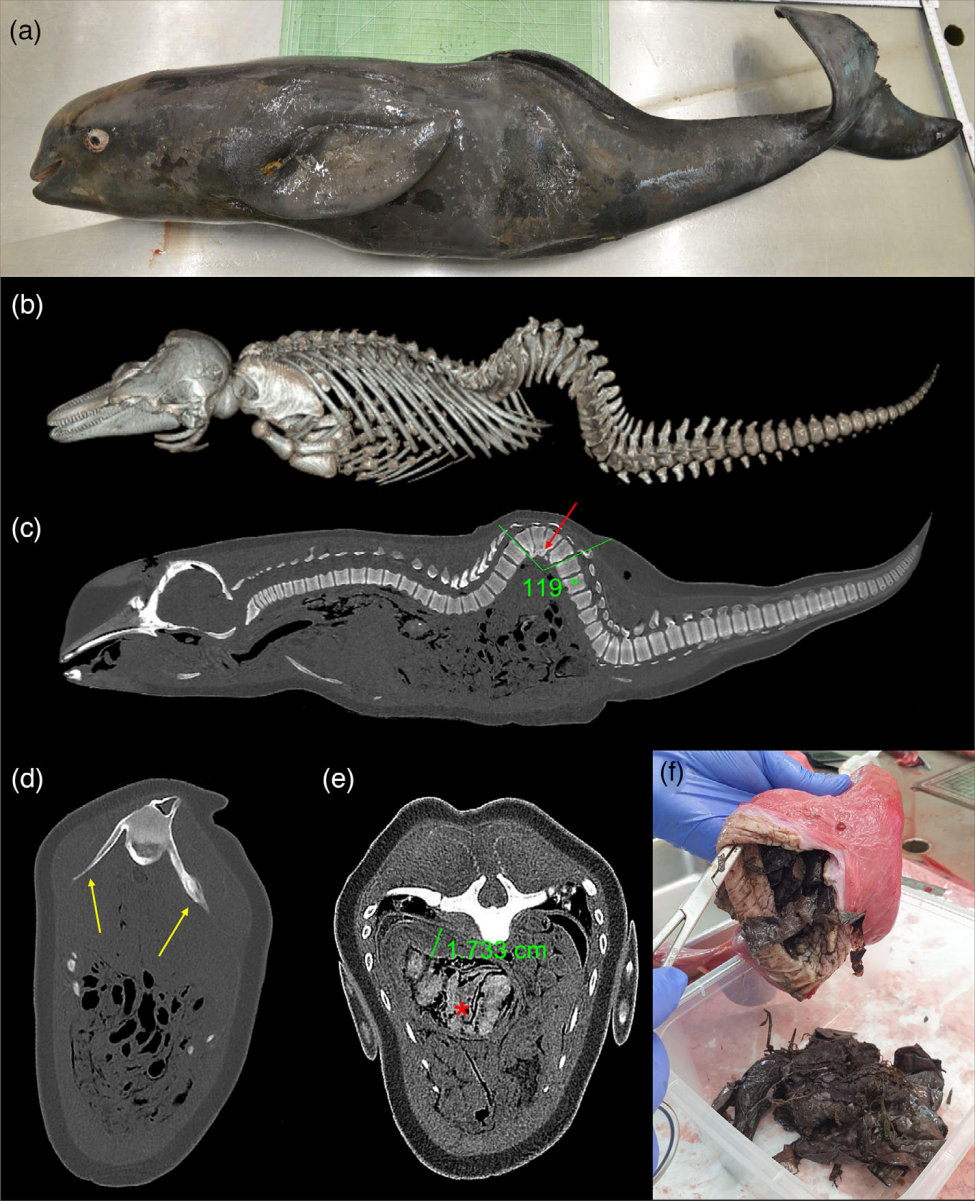
(a) CRI‐11873, an individual narrow‐ridged finless porpoise (NFP). (b) Three‐dimensional (3D) volume‐rendered post‐mortem computed tomography (PMCT) image with bone reconstruction algorithms showcasing kyphoscoliosis in lumbar vertebrae 5–8. (c) Multiplanar reconstructed sagittal PMCT image illustrating kyphoscoliotic vertebrae with an angle of 119°; notable findings include an unilaterally unsegmented bar with hemivertebrae (highlighted by the red arrow). (d) PMCT axial image revealing compression of the longissimus dorsi muscles by the transverse processes of the kyphoscoliotic vertebrae (indicated by orange arrows). (e) PMCT axial image displaying a thickened forestomach wall measuring 1.73 cm. Amorphous contents in the forestomach extending into the oesophagus are evident (marked with an asterisk). (f) Necropsy findings confirm that the amorphous contents observed in the forestomach using PMCT were plastic bags.

Postcranial vertebral formula of CRI‐11873 was C_7_ + T_13_ + L_14_ + Ca_29_. Using PMCT 3D volume‐rendered imaging, kyphoscoliosis was observed in lumbar vertebrae (L) 5–8 (Figure 1b). A unilateral unsegmented bar in L5‐7 resulted in a spinal curvature of 119 degrees (Figure 1c). Intervertebral discs were found between the vertebral bodies, however, degenerated and partially absent. Deformation of the transverse processes of L5‐8, directed ventrally, compressed the longissimus dorsi muscles, creating noticeable muscular imbalances (Figure 1d). No signs of osteophytosis were observed. Bone density measurements were within the normal range throughout the vertebral column. Particularly, the bone density, measured in HU, in L5‐8 was 982, 1005, 1023 and 1076, respectively.

In addition to kyphoscoliosis, PMCT axial images revealed a thickened forestomach wall measuring 1.73 cm, suggestive of gastritis. Amorphous contents in the forestomach extending into the oesophagus were observed (Figure 1e), whereas bowel contents were absent, indicating a potential obstruction of the stomach contents. Gross necropsy revealed that the stomach contents consisted of plastic bag pieces (Figure 1f). The oesophageal mucosa was edematous, and the stomach wall exhibited significant thickening with severe ulceration. Peritoneal fibrosis was observed, along with mild ankylenteron and an enlarged mesenteric lymph node, indicative of peritonitis.

## 4 CRI‐11874 (MALE; 188.3 CM; 59.6 KG) (FIGURE 2a)

**FIGURE 2:**
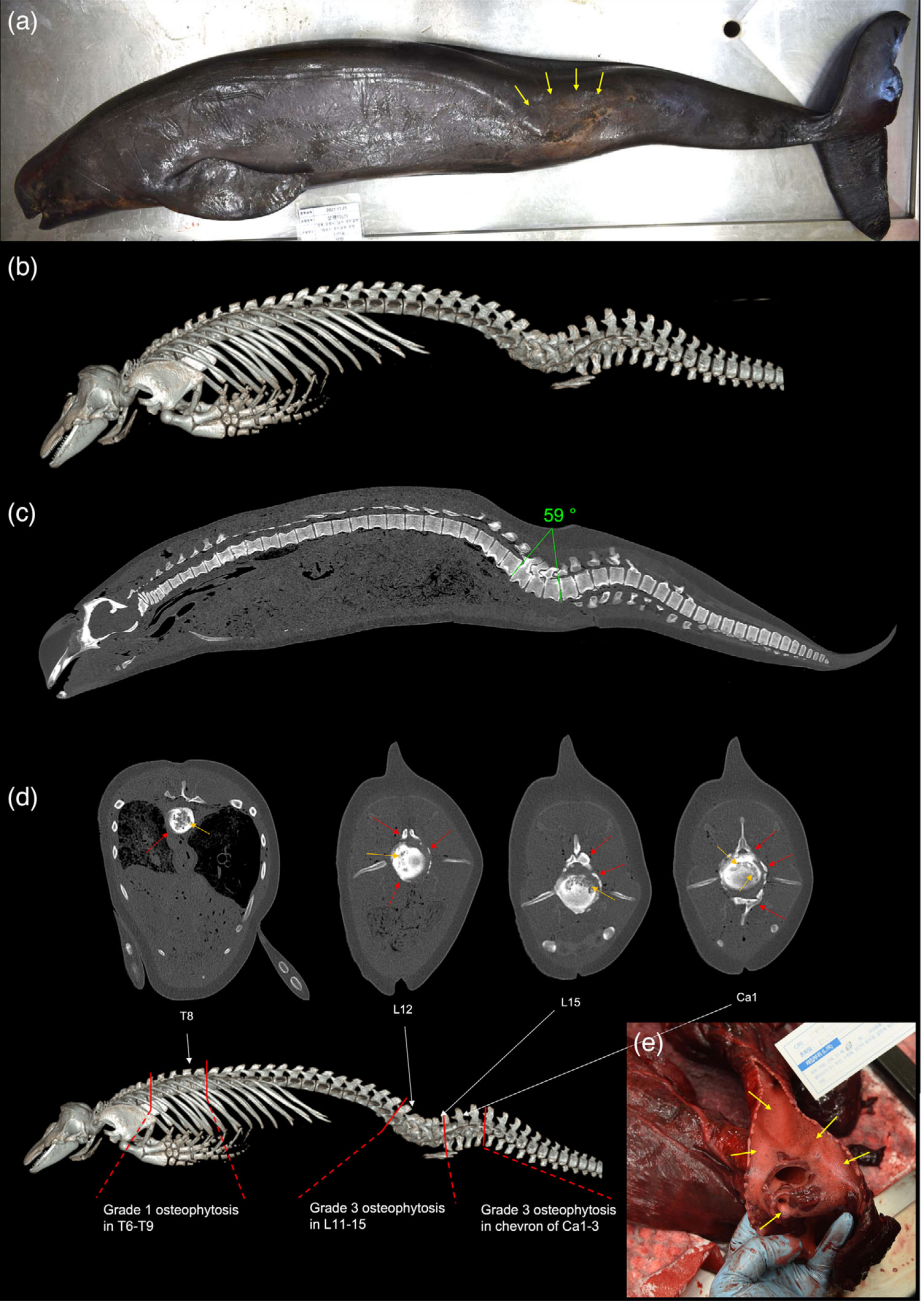
(a) CRI‐11874, an individual narrow‐ridged finless porpoise (NFP), displaying a wide area of scarred skin on the lumbar region (highlighted by orange arrows). (b) Three‐dimensional (3D) volume‐rendered post‐mortem computed tomography (PMCT) image with bone reconstruction algorithms revealing kyphoscoliosis in lumbar vertebrae 11–15 and caudal vertebra 1. (c) Multiplanar reconstructed sagittal PMCT image demonstrating kyphoscoliotic vertebrae with an angle of 59°. (d) PMCT axial images highlighting areas of hyperosteosis (indicated by red arrows) at the pedicle and lamina of thoracic, lumbar and caudal vertebrae, as well as the chevrons, and also the areas of osteoporosis of vertebral bodies (indicated by orange arrow). (e) Trachea and tracheole filled with haemorrhagic foamy fluid (denoted by yellow arrows).

Postcranial vertebral formula of CRI‐11874 was C_7_ + T_12_ + L_15_ + Ca_35_. External examination and necropsy of CRI‐11874 revealed extensive scarring and thinning of the skin in the lumbar region (Figure 2a). PMCT 3D volume‐rendered images and multiplanar reconstructed coronal images disclosed kyphoscoliosis with a curvature of 59 degrees with severe osteophytosis and osteoporosis in L11‐15 and caudal vertebra (Ca) 1 (Figure 2b–d). Multiplanar reconstructed axial images indicated grade 1 osteophytosis in thoracic vertebrae (T) 6–9 and grade 3 osteophytosis in L11‐15 and Ca1, according to the lesion categorization by Nathan (1962). Hyperosteosis was noted at the pedicle and lamina of L14‐15, potentially causing spinal canal compression (Figure 2d). Intervertebral discs were presented but observably compressed in L11‐Ca1. Hyperosteosis was also observed at the chevrons of Ca1‐3 (Figure 2d). The bone density, measured in HU, in T6‐9 was 576, 444, 750 and 429, respectively; in L11‐15 was 397, 693, 469, 373 and 441, respectively; and in Ca1 was 382.

PMCT examination revealed diffuse ground glass opacity patterns in both lungs, along with hyperattenuating focal lesions, suggesting pulmonary oedema and signs of infection. Gross necropsy confirmed the presence of pulmonary emphysema and gas embolism, particularly severe in the right lung. The presence of pulmonary oedema with haemorrhagic foamy fluid was consistent with findings associated with ‘wet drowning’ in cetaceans (IJsseldijk et al., 2021) (Figure 2e). Parasites found in both lungs were identified as *Halocercus sunameri* (*Nematoda*: *Pseudaliidae*) based on morphological analyses (Yamaguti, 1951).

## 5 DISCUSSION

To the best of the authors’ knowledge, this study represents the first‐ever assessment of kyphoscoliosis in cetaceans using PMCT. PMCT, due to its high spatial resolution, contrast resolution and signal‐to‐noise ratio, offers superior diagnostic accuracy for screening osteological anomalies like kyphoscoliosis. It eliminates the need for skeletal excarnation during conventional necroscopic assessment and provides immediate information for evaluating scoliotic spines in their natural position.

**FIGURE 3:**
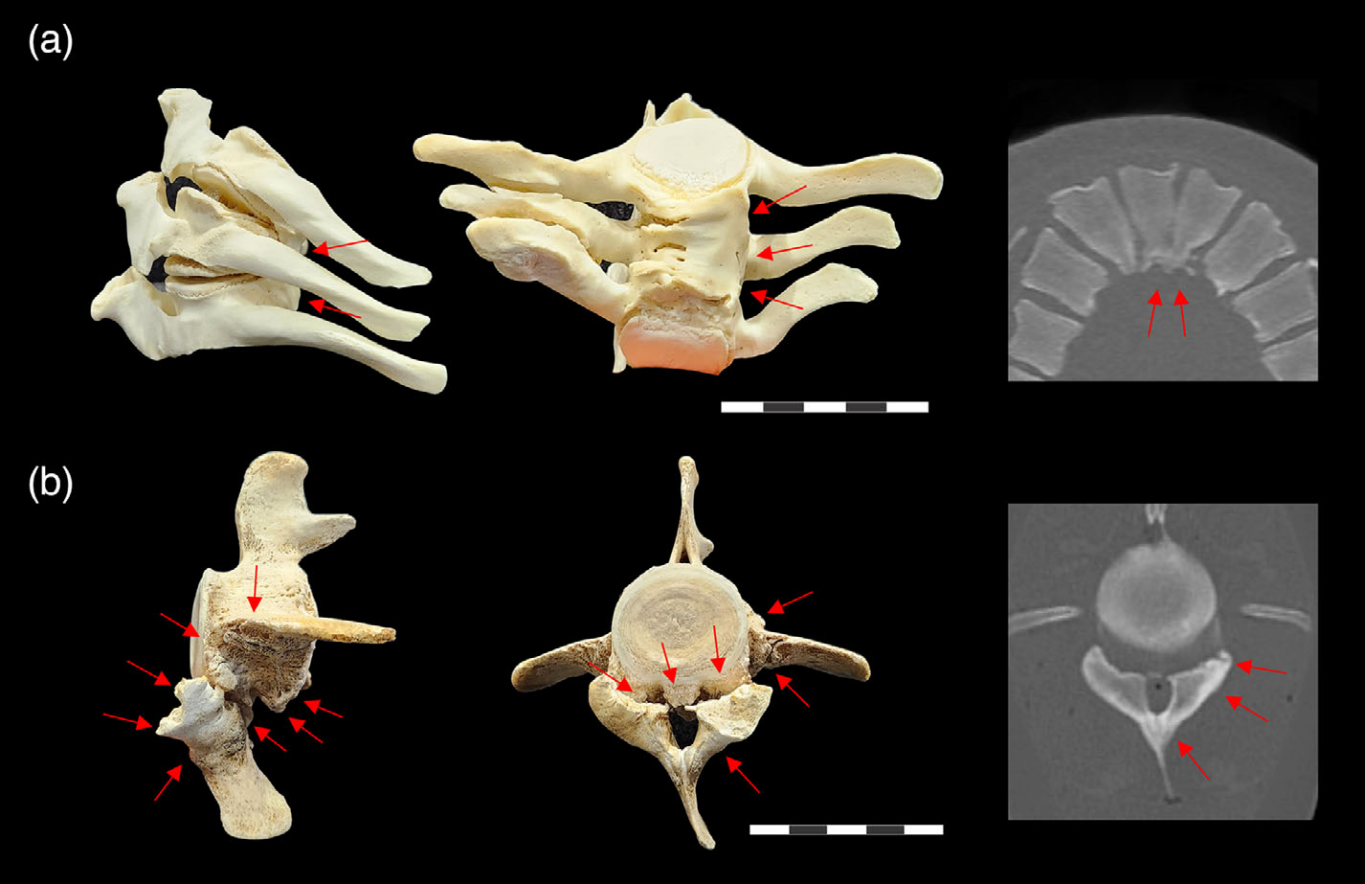
(a) Lumbar vertebrae 5–7 of CRI‐11873. Left: lateral view; middle: ventrodorsal view; right: sagittal section of post‐mortem computed tomography (PMCT). The presence of a unilateral unsegmented bar is evident at the ventral centrum (highlighted by red arrows). (b) Caudal vertebra 3 of CRI‐11874. Left: lateral view; middle: cranial view; right: axial section of PMCT. Osteophytosis is observed on the centrum surface, transverse processes and chevrons (indicated by red arrows). Scale bars = 5 cm.

In CRI‐11873, PMCT and necropsy identified unsegmented bars with hemivertebrae in L5‐7 (Figure 3a). The occurrence of kyphoscoliosis with unsegmented bar in marine mammals has received limited research attention. To date, DeLynn et al. (2011) have reported the only case of congenital kyphoscoliosis in a bottlenose dolphin, which has demonstrated unsegmented ribs and cervical vertebrae. Despite locational differences in the affected sites, both specimens from DeLynn et al. (2011) and CRI‐11873 have shown characteristic patterns of congenital skeletal deformity, including defects in formation and segmentation. The unsegmented bar is one of the representative anomalies caused by impaired embryonic development in the early stage of pregnancy (Giampietro et al., 2013). It is caused by unilateral disruption of the segmentation of the primary vertebral mesenchyme, resulting in the connection of two or more adjacent vertebrae. As the opposite side of the vertebrae, on which the growth zones and part of the intervertebral disc are preserved, develops normally, abnormal spinal curvature occurs, resulting in congenital spinal deformity. The presence of a unilateral unsegmented bar with characteristic conformation led to the classification of the kyphoscoliosis as congenital in CRI‐11873.

Infanticidal events could be another cause of kyphoscoliosis in young individuals. This behaviour is considered an expression of conflict in cetaceans to protect prey resources and mating access (López et al., 2018). Robinson (2014) reported a case of potential infanticide‐inflicted kyphoscoliosis in a calf bottlenose dolphin. The calf was found stranded and dead approximately half‐year after an attempted infanticidal event. Acute kyphoscoliosis was revealed during gross necropsy with observed remodelling of the lateral spinal processes and was believed to be acquired from trauma as a neonate. However, during PMCT scanning of CRI‐11873, no bone remodelling and realignment, an essential process to repair damaged bone, was observed. No post‐traumatic scars were found during necropsy. With the presence of unilateral unsegmented bar with hemivertebrae in CRI‐11873, kyphoscoliosis as a result of infanticidal event could be ruled out in this case.

Although kyphoscoliosis may not be fatal in cetaceans, previous reports have documented cetaceans with kyphoscoliosis living up to 39 years (Galatius et al., 2009). However, it may impede effective movement, a fundamental ability for interacting with conspecifics and hunting prey, potentially reducing survival and reproductive success. In CRI‐11873, the longissimus dorsi muscles were noticeably compressed by the transverse processes of kyphoscoliotic L5‐8 (Figure 1d), potentially limiting the vertical oscillation of the caudal peduncle and flukes (Strickler et al., 1980; Viglino et al., 2014). Together with unbalanced and compressed epaxial muscles and reduced joint motion in the affected vertebrae, limited body movements due to spinal deformity could have affected this individual's ability to hunt active prey.

The NFPs inhabit the east Asian coast, a geographic hotspot of anthropogenic threats (Walther et al., 2020; Xiong et al., 2018; Yuen et al., 2023). Rapidly growing anthropogenic activities are influencing NFPs in this area. The human‐induced pressure of plastic litter has become a major threat to the marine environment (Villarrubia‐Gómez et al., 2018). In recent years, scientific attention has turned to the discovery of plastics in cetaceans (Besseling et al., 2015; Curran et al., 2014; Lusher et al., 2015; Xiong et al., 2018). Several studies have reported an increasing trend in cetaceans interacting with (Rodríguez et al., 2023), or even ingesting, plastic bags (Đuras et al., 2021; Sá et al., 2023). In the present report, our team also discovered that CRI‐11873 may have mistakenly ingested the plastic bags. It is believed that the presence of plastics in cetaceans’ digestive tracts is owing to misinterpretation as natural prey or accidental ingestion (Đuras et al., 2021; Sá et al., 2023). Nonetheless, ingested plastics can lead to gastrointestinal disorders such as impacted stomach, ulcerative gastritis and ultimately contributing to the animals’ death.

In CRI‐11874, osteophytosis, osteoporosis and hyperosteosis were observed in thoracic, lumbar and caudal vertebrae through PMCT and necropsy (Figure 3b). Given the adult life stage of this individual (Lee et al., 2013) with the existence of osteoporosis, the kyphoscoliosis was possibly attributed to degeneration. In the early stages of spinal degeneration, the formation of osteophytes in vertebrae may provide stabilization for mildly wedged segments, counteracting spinal instability. As degeneration progresses, asymmetric osteophyte growth can distort spinal stability, increasing the likelihood of degenerative scoliosis and limiting bending movements (Zhu et al., 2017). Additionally, antemortem traumatic events were suggested as a contributing factor, as evidenced by the broad scarring and thinning of lumbar skin observed during necropsy. Altogether, while the degenerative change of the skeletal system including the osteoporosis weakened the intensity of the system, traumatic events occurred and possibly contributed to the kyphoscoliosis. Nonetheless, the cause of death in this individual was presumed to be aspiration pneumonitis secondary to drowning (Reijnen et al., 2019). The reduced spinal motion range due to kyphoscoliosis has potentially contributed to its demise.

NFPs are known to be hard to detect due to their small body size, small group sizes, tendency to avoid boats and unobtrusive surface behaviours. The diagnosis of kyphoscoliosis, as well as understanding the associated impact on survival and socio‐ecological behaviour, can be even more challenging and, therefore, remains poorly understood in NFPs. The long‐term survival of cetaceans with kyphoscoliosis may depend on various factors, including, but not limited to, the age and level of maternal dependence of the affected calf, the nature of the kyphoscoliosis (congenital, acquired, or degenerative), the severity of the condition and any resulting complications (Bertulli et al., 2015; DeLynn et al., 2011). Nonetheless, the most influential factor that affects a kyphoscoliotic individual's survival remains the degree of anomaly that impedes their mobility. In Indo‐Pacific and Atlantic humpback dolphins, Weir and Wang (2016) reported that impaired mobility not only affects an individual's ability to capture prey and avoid predators but also makes them more vulnerable to anthropogenic activities, such as vessel collisions, due to reduced swimming speed and longer surfacing time. In white‐beaked dolphins, individuals with kyphoscoliosis may be more prone to being caught in gillnets, longline fisheries and trawl nets (Bertulli et al., 2015).

Interactions of kyphoscoliotic cetaceans with conspecifics have also been occasionally reported, although the available data is currently limited to bottlenose dolphins. Haskins and Robinson (2007) reported that a female bottlenose dolphin with lordosis gave birth to two calves over a span of seven years, as observed through photo‐identification. Additionally, Wilson and Krause (2013) documented a case in which a bottlenose dolphin with kyphoscoliosis repeatedly interacted, socialized, and milled with a group of sperm whales. These findings may suggest that despite impaired mobility, the influences of kyphoscoliosis on cetaceans’ socio‐ecological behaviour may be relatively minor than expected.

In human medicine, the Cobb angle assessment (Cobb, 1947) is the gold standard for evaluating kyphoscoliosis, measuring the angulation of the most tilted vertebrae in coronal plain radiographs or CT images. Cobb angle assessment provides important information leading to justified clinical decisions such as progression, as well as physiotherapeutic and surgical interventions. Although standardized measurements for kyphoscoliosis assessment in cetaceans are currently lacking in the literature, our group applied an analogous method of Cobb angle assessment in sagittal CT images to measure spinal curvature in both NFPs. Cobb angle can be measured after finding the most‐tilted vertebral body above and below the main curvature apex of each NFP vertebrae. The angle formed by the extension lines from each vertebral body, as marked in Figures 1c and 2c was measured. The results aligned with the diagnoses made by the board‐certified veterinarians (SWK and KL). Future research with a larger sample size is warranted to establish standardized radiological measurements for diagnosing kyphoscoliosis in cetaceans.

For assessing the degree and aetiology of spinal deformities in cetaceans, the use of PMCT is essential. When necropsy is used as a sole methodology for determining spinal deformities, examination of vertebrae can only occur after complete defleshing (Figure 3), resulting in drawbacks such as time consumption, loss of in situ location information of the bones and potential loss of small bone fragments. PMCT enables quantitative analyses, including analogous Cobb angle assessment and bone density analysis, and allows for the CT data storing into a database regardless of the passage of time, such as picture archiving and communication system, and could be retrieved at will. This case report not only presents PMCT data on kyphoscoliosis in NFP for the first time but also describes and compares two cases of spinal deformities caused by congenital and acquired factors. It serves as a valuable reference for future studies investigating the aetiology of deformations based on PMCT.

## 6 CONCLUSION

This case report is the first documented instance of kyphoscoliosis in *Phocoenidae*, specifically in NFP, utilizing PMCT to provide additional information alongside gross necropsy. The causes of kyphoscoliosis in the calf and adult individuals were classified as congenital and degenerative, respectively. This report enhances our understanding of potential causes of kyphoscoliosis in NFP based on PMCT data analysis.

## AUTHOR CONTRIBUTIONS

*Conceptualization*: Adams Hei Long Yuen and Sang Wha Kim. *Data curation*: Sang Wha Kim, Kyunglee Lee, Adams Hei Long Yuen, Min Ju Kim, Cherry Tsz Ching Poon and Young Min Lee. *Investigation*: Sang Wha Kim, Kyunglee Lee, Sung Bin Lee, Won Joon Jung, Young Min Lee, Su Jin Jo, Mae Hyun Hwang and Jae Hong Park. *Writing – original draft*: Adams Hei Long Yuen and Sang Wha Kim. *Writing – review and editing*: Sang Wha Kim, Adams Hei Long Yuen and Sib Sankar Giri. *Resources*: Sang Wha Kim and Kyunglee Lee. *Supervision*: Seung Hyeok Seok and Se Chang Park.

## CONFLICT OF INTEREST STATEMENT

The authors declare no conflicts of interest.

### ETHICS STATEMENT

The authors confirm that the ethical policies of the journal, as noted on the journal's author guidelines page, have been adhered to and the appropriate ethical review committee approval has been received. The US National Research Council's guidelines for the Care and Use of Laboratory Animals were followed.

### PEER REVIEW

The peer review history for this article is available at https://www.webofscience.com/api/gateway/wos/peer-review/10.1002/vms3.1386.

## Data Availability

Data available on request from the authors.
